# Long-Term Effects of Myoinositol on Behavioural Seizures and Biochemical Changes Evoked by Kainic Acid Induced Epileptogenesis

**DOI:** 10.1155/2019/4518160

**Published:** 2019-02-28

**Authors:** Lia Tsverava, Manana Kandashvili, Giorgi Margvelani, Tamar Lortkipanidze, Georgi Gamkrelidze, Eka Lepsveridze, Merab Kokaia, Revaz Solomonia

**Affiliations:** ^1^Institute of Chemical Biology, Ilia State University, 3/5 K. Cholokashvili Avenue, 0162 Tbilisi, Georgia; ^2^I. Beritashvili Center of Experimental Biomedicine, 14 L. Gotua Street, 0160 Tbilisi, Georgia; ^3^Epilepsy Centre, Department of Clinical Sciences, Lund University Hospital, Lund, Sweden

## Abstract

Epilepsy is one of the most devastating neurological diseases and despite significant efforts there is no cure available. Occurrence of spontaneous seizures in epilepsy is preceded by numerous functional and structural pathophysiological reorganizations in the brain—a process called epileptogenesis. Treatment strategies targeting this process may be efficient for preventing spontaneous recurrent seizures (SRS) in epilepsy, or for modification of disease progression. We have previously shown that (i) myoinositol (MI) pretreatment significantly decreases severity of acute seizures (status epilepticus: SE) induced by kainic acid (KA) in experimental animals and (ii) that daily post-SE administration of MI for 4 weeks prevents certain biochemical changes triggered by SE. However it was not established whether such MI treatment also exerts long-term effects on the frequency of SRS. In the present study we have shown that, in KA-induced post-SE epilepsy model in rats, MI treatment for 28 days reduces frequency and duration of behavioural SRS not only during the treatment, but also after its termination for the following 4 weeks. Moreover, MI has significant effects on molecular changes in the hippocampus, including mi-RNA expression spectrum, as well as mRNA levels of sodium-MI transporter and LRRC8A subunit of the volume regulated anionic channel. Taken together, these data suggest that molecular changes induced by MI treatment may counteract epileptogenesis. Thus, here we provide data indicating antiepileptogenic properties of MI, which further supports the idea of developing new antiepileptogenic and disease modifying drug that targets MI system.

## 1. Introduction

Epileptogenesis is a dynamic and multifactorial process of molecular, cellular, and functional reorganization in the brain that follows the precipitating events or insults that lead to epilepsy—a disease which is characterized by spontaneous recurrent seizures (SRS) [1]. Approximately 1% of the human population in the world suffer from epilepsy. Currently available antiepilepsy drugs (AEDs) offer only symptomatic relief by suppressing SRS, but they are not able to prevent or cure epilepsy. In addition 20%–30% of the patients are refractory to AEDs [1–3]. It has been proposed that treatment strategies that could interfere with the process of epileptogenesis would provide significant benefit by preventing or modifying the disease. Unfortunately, at present there are no drugs available that could effectively prevent the process of epileptogenesis or modify the disease in humans or experimental animals [1–3]. The antiepileptogenesis treatment may also exert disease modification effect, which means that if treatment does not fully prevent the development of the disease, it will nevertheless weaken its course in terms of SRS frequency and/or severity [2].

In Chinese and Tibetan folk medicine, some native plants of the* Ranunculacae *family (that contains plant* Aquilegia *vulgaris) are widely used as antiepileptic and soporific medication [4]. In our early studies, we have shown that water extract of* Aquilegia vulgaris* contains compounds affecting *γ*-aminobutyric acid- (GABA-) A receptor ligand binding in vitro. They were identified as myoinositol (MI) and sleep-inducing lipid oleamide. MI completely inhibits ^3^H-muscimol (a GABA-A receptor agonist) binding in rat brain-derived cell membranes, while oleamide increases ^3^H-Flunitrezepam (a specific ligand for the GABA-A receptor benzodiazepine site) binding by approximately a factor two [4]. GABA-A receptor is a ligand-gated chloride channel that mediates synaptic inhibition in the central nervous system. A number of antiseizure drugs have agonistic effects on GABA-A receptors [5]. Moreover, mutations in GABA-A receptor subunits have been shown to be associated with human epilepsy [6].

In the previous series of experiments, we demonstrated that MI pretreatment significantly decreases severity of acute seizures induced either by pentylentetrazolium (PTZ) or by kainic acid (KA) in experimental animals [7, 8]. In the following series of experiments MI treatment was initiated and lasted for 28 days, only after status epilepticus (SE) was induced by KA. The KA-induced SE leads to generation of SRS after a certain latent period during which the epileptogenesis is thought to take place [reviewed in [9]]. The experimental samples for biochemical analysis were taken 1 day after the termination of MI treatment (29 days after SE). In these samples, we have found that MI treatment attenuated biochemical changes taking place during the epileptogenesis. Namely, KA-induced epileptogenesis was characterized by a strong decrease (>60%) in the levels of AMPA-glutamate receptor GLUR1 subunit, calcium-calmodulin dependent protein kinase II (CaMKII), and gamma-2 subunit of GABA-receptor in the hippocampus and this decrease was nearly completely reversed by MI daily treatment [10, 11]. We have suggested that the effects of MI at least partially should be mediated by its osmolyte properties [10–12].

In the present study, we hypothesized that the MI-induced normalization of biochemical alterations during epileptogenesis can lead to disease modification and decrease SRS frequency and duration in the chronic phase of epilepsy. In particular, we investigated whether 4 week post-SE MI administration exerted long-term effects on the frequency and duration of SRS after the termination of the treatment, and this was accompanied by the molecular changes in the hippocampus. Rats were treated by MI for the first 28 days after KA-induced SE and decapitated after additional 4 weeks for further biochemical analysis. During the entire period, animals were continuously video monitored for frequency and duration of behavioural SRS.

The rationale of biochemical studies has been the following: (i) MI is accumulated into the cells by sodium-myoinositol transporter (SMIT). SMIT mRNA is upregulated in the rat brain at least for 12h after the onset of KA-induced seizures [13]; (ii) leucine-rich repeat-containing 8A (LRRC8A) is essential component of volume regulated anionic channel (VRAC) [14, 15]. The main function of this channel is Cl-transport [reviewed in [16]] but they are permeable also to large anionic excitatory amino acids (EAA) including glutamate. It is suggested that VRACs represent a pathway through which astrocytic EAAs might be released back into the extracellular space and increase neuronal excitability [reviewed in [17]]; (iii) epileptogenesis is accompanied by specific changes in gene expression patterns [for review [18]]. Well- known regulators of coordinated changes in gene expression are micro-RNAs (miRNA). Deregulation of diverse brain-specific miRNAs was observed in animal models of epilepsy as well as in patients with epilepsy [see for review [19]]; [iv] epileptogenic injuries trigger sterile neuroinflammation—a complex phenomenon involving the activation of the innate immune system by damage-associated molecular patterns (DAMPs) [20]. The High Mobility Group Box 1 (HMGB1) is a prototypical member of the DAMP family. It should be noted that some of these biochemical changes (e.g. LRRC8-A expression) have never been studied in epileptogenesis or 8 weeks after KA-induced SE (e.g., SMIT expression)

In support of our hypothesis, we demonstrate that (i) frequency and duration of SRS are significantly reduced in MI-treated animals as compared to KA+Saline (KA+SAL) group during 8 weeks after SE; (ii) expression of SMIT is upregulated in the hippocampus in KA+SAL and in KA+MI groups, with the significantly highest level in MI-treated group; (iii) expression of LRRC8A is increased only in KA+SAL group whereas in MI-treated animals LRRC8A maintains the same control level; (iv) various changes in mi-RNA spectrum have been observed in the KA+SAL and KA+MI groups comparing to control groups. It is noteworthy that some of the miRNAs in MI-treated group remain on the same level as in the control group.

## 2. Materials and Methods

### 2.1. KA-Induced SE

Male Wistar rats, 2.5–3 months of age, received a single intraperitoneal injection (IP) of KA (10mg/kg, Abcam) dissolved in saline. After injection, each animal was placed into an individual plastic cage for observation for 4 hours. Seizures were scored according to a modified Racine scoring system from 0 to 6: (0) no motor seizures; (1) freezing, staring, mouth, or facial movements; (2) head nodding or isolated twitches, rigid posture; (3) tail extension, unilateral–bilateral forelimb clonus; (4) rearing, in which the mice sit in an immobile state on their rear haunches with one or both forepaws extended; (5) clonic seizures, loss of posture, jumping, and forepaws extended; (6) tonic seizures with hind limb extension [21, 22]. Animals with seizures grades 4-6 were selected for further research. Selected animals exhibited seizures at least for 60 minutes during 4 hour observation period—which is enough to induce epileptogenesis and consequently epilepsy for this type of treatment [2].

Half of selected KA treated rats were treated with MI (30mg/kg, KA+MI group) and another half with saline (0.9% NaCl sterile solution, 1 mL/kg KA+SAL group). IP injections (twice per day) started 4 hours following KA treatment and continued for 28 days.

Control animals were treated with saline and then divided into two groups: one group received twice daily saline injections (0.9% NaCl sterile solution, 1 mL/kg, and CON+SAL group) and the other group twice daily MI injections (30mg/kg, CON+MI group). Injections lasted for 28 days, like in case of KA treated animals.

**Figure 1:**
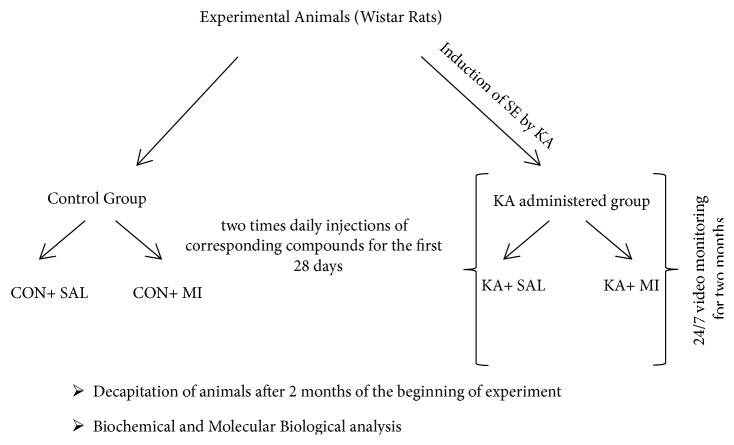
The diagram of experimental design.

The diagram of experimental design is provided in Figure 1.

### 2.2. Video-Monitoring

Animals were housed individually and maintained under 12 h light/12 h dark cycle. Animal behaviour was 24/7 monitored by closed-circuit infrared video cameras (IR_IP66_ HIK VISION). Digital video files were recorded with Digital Video Recorder (HIK VISION, DS-7316HLS) and kept on removable high capacity hard disks. Recordings were reviewed visually offline (played at 4X speed) to detect any behavioural seizure activity according to a modified classification scale described above [21, 22]. Only seizures of 4-6 grades were evaluated, since lower grade seizures can easily be confused with normal behaviour. Animals were decapitated on 56^th^ day after the beginning of experiments. Hippocampus and neocortex were extirpated and frozen immediately on dry ice. In parallel to decapitation, blood was collected in EDTA containing tubes and plasma was isolated according to standard procedures, aliquoted, and stored at –80°C until assayed.

### 2.3. RNA Isolation

For miRNA profiling RNA was isolated from hippocampus by miRNeasy Mini Kit (Qiagen, 217004). This kit is useful for the isolation of total RNA including small RNAs. The concentration of RNA was measured by absorbance at wavelength 280/260nm on Nanodrop.

The RNA isolation for miRNA quantitative measurement, as well as for SMIT and LRRC8A mRNA measurements, was carried out by the same miRNeasy Mini Kit.

### 2.4. *μ*Paraflo™ MicroRNA Microarray Assay

miRNA profiling experiments were done on hippocampi of three groups of rats: CON+SAL, KA+SAL, and KA+MI, with 5 animals in each group. miRNA profiling was performed using a service provider (LC Sciences). One *μ*g of total RNA was used for analysis. Samples were 3'-extended with a poly(A) tail using poly(A) polymerase. An oligonucleotide tag was ligated to the poly(A) tail for later fluorescent dye staining. Hybridization was performed overnight on a *μ*Paraflo microfluidic chip using a micro-circulation pump (Atactic Technologies) [23, 24]. Each detection probe on the microfluidic chip consisted of a chemically modified nucleotide coding segment, complementary to species specific 991 target miRNAs (from miRBase version 21, http://mirbase.org) in triplicates or other RNA (control) and a spacer segment of polyethylene glycol, to extend the coding segment away from the substrate. Detection probes were made by* in situ* synthesis using photogenerated reagent chemistry. The hybridization melting temperature was balanced by chemical modifications of the detection probes. Hybridization used 100 *μ*L 6xSSPE buffer (0.90 M NaCl, 60 mM Na_2_HPO_4_, 6 mM EDTA, and pH 6.8) containing 25% formamide at 34°C. After RNA hybridization, tag-conjugating Cy3 dye was circulated through the microfluidic chip for dye staining. Fluorescence images were collected using a laser scanner (GenePix 4000B, Molecular Device) and digitized using Array-Pro image analysis software (Media Cybernetics). Data were analyzed by first subtracting the background and then normalizing the signals using a LOWESS (locally weighted regression) filter [25].

### 2.5. miRNA Measurement

For the measurement of the selected mi-RNA (miRNA 6216), samples from the following 4 groups of rats were used: CON+SAL, CON+MI, KA+SAL, and KA+MI. The RNA fractions from hippocampus and neocortex were reverse transcribed by Taqman MicroRNA Reverse Transcription Kit (Thermo Fisher Scientific 4366596). The amount of miRNA 6216 was measured by TaqMan™ assay kit from Thermo Fisher (Assay ID-471281_mat, Thermo Fisher Scientific) using Step One Real-Time PCR System (Applied Biosystems) and normalized to the amount of the two following miRNAs: miR-23b-3p (Assay ID 245306_mat, Thermo Fisher Scientific) and miR-361-5p (Assay ID 000554,Thermo Fisher Scientific. For selection of housekeeper microRNAs see “Results”). The comparative CT (ΔΔCT) method was used to determine the relative target quantity in samples [26]. The same fraction of hippocampal RNA, isolated from not-treated rats, was used in all experiments as a reference sample. Triplicates were included for each reaction.

### 2.6. SMIT and LRRC8A mRNA Measurement

Complementary DNA (cDNA) from the RNA fractions of hippocampus and neocortex was synthesized by RETROscript Reverse Transcription kit for RT_PCR (Thermo Fisher Scientific AM 1710). Relative SMIT and LRRC8A cDNA copy number was determined by real-time PCR using the Step One Real-Time PCR System (Applied Biosystems) with the SYBR Green detection method and was normalized to the beta-actin. The following primers were used:

1. LRRC8A: Forward primer: GCACCAACAGCCAACACAAAG
2. Reverse primer: GGAGTCGTTGCAGGAGTCTT
3. SMIT: Forward primer: TGG TGA CGA AGG AGA GTT GC
4. Reverse primer: AGG TTG GAG CCC CTT AAT GC
5. *β*-actin: Forward primer: 5′-CTACAATGAGCTGCGTGTGG-3′
6. Reverse primer 5′: CTCCGGAGTCCATCACAATG-3.

### 2.7. Subcellular Fractionation

HMGB1 was determined in the hippocampus and neocortex as well as in the blood plasma samples from the following 4 groups of rats: CON+SAL, CON+MI, KA+SAL, and KA+MI. Brain tissue samples were rapidly homogenized in 20mM Tris-HCl (pH 7.4), 0.32M sucrose, 1mM Methylendiamintetraacetic acid, 1mM sodium orthovanadate, 10mM sodium pyrophosphate, 0.5mM ethylene glycol-bis (2-aminoethylether)-N,N,N',N'- tetraacetic acid, and a cocktail of protease inhibitors (Sigma, P8340).

### 2.8. Protein Determination

The protein concentration was determined in brain tissue homogenate fraction as well as in plasma fractions in quadruplicate, using a micro bicinchoninic acid protein assay kit (Pierce).

### 2.9. Electrophoresis and Western Immunoblotting

Equal volume aliquots, containing 30 *μ*g protein, were applied to the sodium dodecyl sulphate (SDS) gels and electrophoresis and Western blotting were carried out as described previously [12]. After the protein had been transferred onto nitrocellulose membranes, the membranes were stained with Ponceau S solution and analyzed with Image J software (https://imagej.net/ImageJ) to confirm uniform gel loading and transfer. Standard immunochemical procedures were carried out using primary antibodies against HMGB1 (Abcam, ab18256) and peroxidase-labelled secondary antibodies and Super-Signal West Pico Chemiluminescent substrate (Pierce) [12]. The optical densities of bands, corresponding to the HMGB1 were measured using Lab Works 4.0 (UVP). The autoradiographs were calibrated by including in each gel four standards of homogenate or plasma samples (15, 30, 45, and 60 *μ*g of corresponding total protein) obtained from the control rats. Optical density was proportional to the amounts of HMGB1 in either brain homogenates or plasma (see Figure 7). For the data analysis optical density of each sample band was divided by optical density of the band for 30 *μ*g of protein standard [12] to give “relative amount of protein”.

Data were not normalized with respect to any other housekeeping protein in brain tissue samples, because it cannot be guaranteed that such proteins are not affected by KA treatment [27, 28]. Gel loading was therefore controlled by Ponceau S staining, Image J software analysis, and calibration with protein standards.

### 2.10. Statistical Analysis

#### 2.10.1. Behavioural Experiments

The data of seizure frequency and duration were analyzed by one-way ANOVA, with factor-treatment (KA+SAL or KA+MI). Analysis was done on the one hand for 8 weeks in total and, on the other hand, for the first the last 4 weeks separately. In case of significant effect in ANOVA, planned comparisons were done by two-tailed t-test.

#### 2.10.2. miRNA-6216 and SMIT and LRRC8A mRNAs

Study of changes in the amounts of miRNA 6216 and SMIT and LRRC8A mRNAs was carried on the following groups of rats: CON+SAL, CON+MI, KA+SAL, and KA+MI. In sum, data were obtained from 36 rats (9 animals per group) from three standard experimental series. To eliminate unavoidable variations between experimental series, the RNA data were divided on the mean of the corresponding experimental series. The data were then analyzed by one-way ANOVA, with factor-treatment (CON+SAL, CON+MI, KA+SAL, and KA+MI). In case of significant effect in ANOVA, planned comparisons were done by two-tailed t-test.

HMGB1. The amount of HMGB1 was determined in the following groups of rats: CON+SAL, CON+MI, KA+SAL, and KA+MI. Each group consisted of 5 animals from two series of experiments. The statistical analysis was done as in case of miRNA and mRNA experiments.

## 3. Results

### 3.1. Effect of MI Treatment on SRS

#### 3.1.1. SRS Frequency

**Figure 2:**
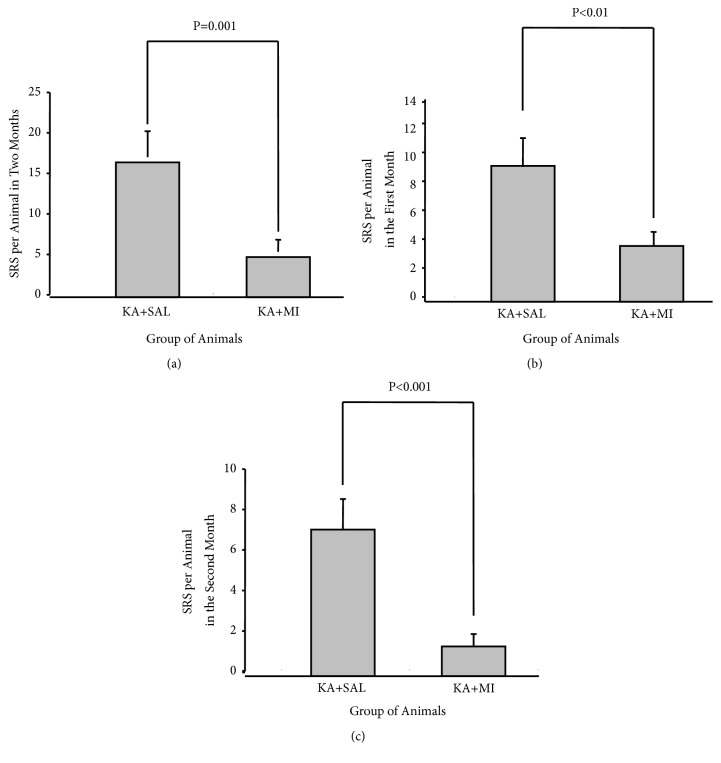
Mean number of SRS per animal during all 8 weeks (a); during MI and SAL treatment (I-IV weeks) (b); after MI and SAL treatment termination (V-VIII weeks) (c).

One-way ANOVA analysis revealed a significant effect of MI treatment on the mean number of SRS per animal during 8 weeks of observation (F1,86=12.25, P=0.001). The mean number of SRS per animal was nearly three times less in MI-treated group of rats as compared to the KA+SAL group (T = 3.50 P-Value = 0.001 85 DF, Figure 2(a)).

Analyzing data by two separate 4-week time frames has shown that the effect of MI treatment was significant for both periods—for the first 4 weeks as well as for the following 4 weeks, when the MI treatment had already been discontinued (correspondingly F 1,86= 7.16, P=0.009 and F 1,86=14.59, and P<0.0001). The mean number of SRS per animal was significantly lower in MI-treated group for both periods (correspondingly T = -2.68 P = 0.009 85 DF and T = 3.82 85 DF, P< 0.0001). These differences were even more pronounced for the second 4-week period (Figures 2(b) and 2(c)). Thus 4-week MI treatment after KA-induced SE has long-term suppressing effects on the SRS frequency at least for 4 weeks after the termination of the treatment.

#### 3.1.2. SRS Duration

**Figure 3:**
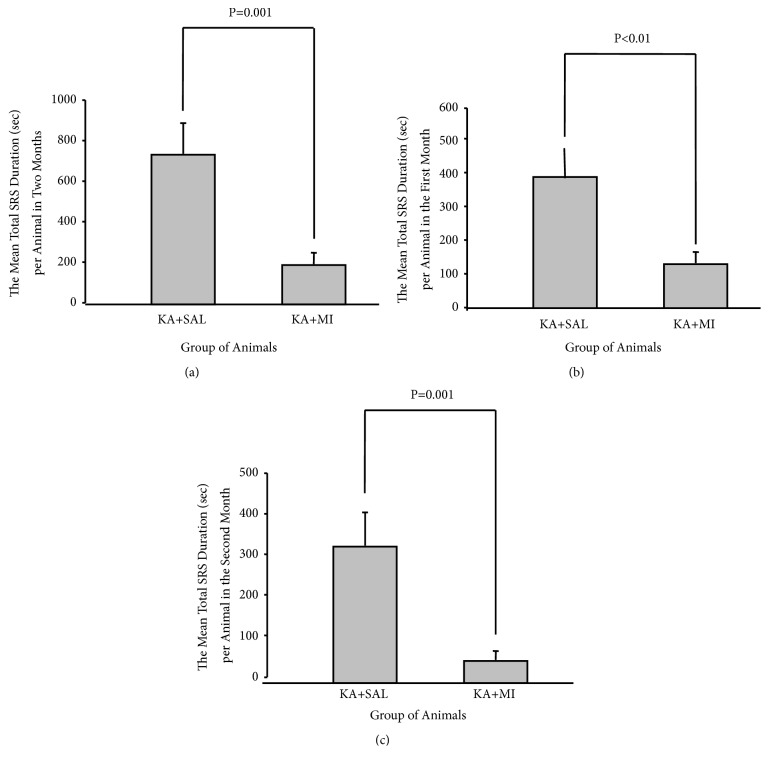
Mean total duration (seconds) of SRS per animal during all 8 weeks (a); during MI and SAL treatment (I-IV weeks) (b); after MI and SAL treatment termination (V-VIII weeks) (c).

MI treatment for 4 weeks had also a significant effect on total time that animals spent in seizure (sum of all SRS durations) during all 8 weeks of experimental period (one-way ANOVA, F1,86=13.41 P<0.0001). The mean total SRS duration (sum of all SRS durations divided on the number of animals in the group) in MI-treated rats was significantly less comparing to KA+SAL group (T= 3.66 85 DF P < 0.0001, Figure 3(a)). Data analysis of the first and the second 4-week periods revealed that effect was significant not only during MI treatment (the first 4 weeks, F1,85= 7.26 P=0.009) but also after its termination (the second 4 weeks, F1.85=13.36, and P<0.0001). For both periods the mean total duration of SRS was significantly less in MI-treated group as compared to the KA+SAL group (for weeks I-IV T= 2.69 85 DF, P= 0.009 and for weeks V-VIII T= 3.65 85 DF P <0.001 Figures 3(b) and 3(c)). The difference between the groups was more expressed for the weeks V-VIII.

### 3.2. Effects of MI Treatment on Biochemical Changes

#### 3.2.1. SMIT mRNA Levels

**Figure 4:**
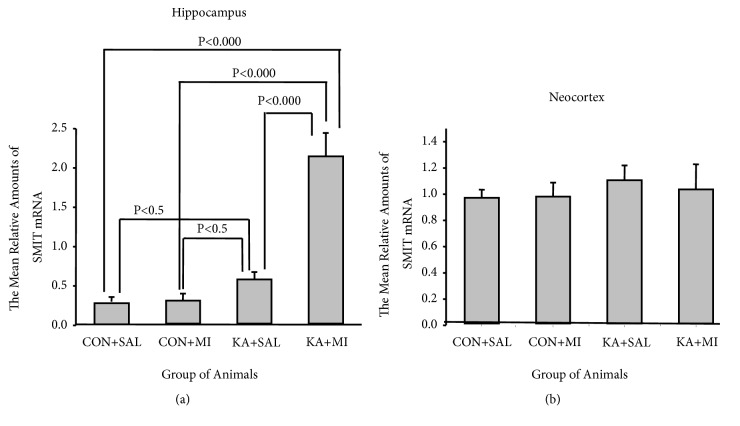
The mean relative levels of SMIT mRNA in hippocampus (a) and in neocortex (b).

In the hippocampus, SMIT mRNA levels were significantly changed by experimental conditions (one-way ANOVA F 3,35=30.11 P<0.0001). The highest mean levels of SMIT mRNA were observed in KA+MI group, which significantly exceeded other three groups (KA+MI vs KA+SAL T = 5.09 16 DF P < 0.001; KA+MI vs CON+SAL T = 6.00 16 DF P< 0.001; KA+MI vs CON++MI T = 6.17 16 DF P< 0.001). The mean levels of SMIT mRNA in KA+SAL group were significantly higher than in CON+SAL group (T= 2.30, 16 DF P= 0.035) and CON+MI group (T = 2.80 16 DF P = 0.013), Figure 4(a).

In the neocortex no significant difference was observed in SMIT mRNA levels between the groups (Figure 4(b)).

#### 3.2.2. LRRC8A

**Figure 5:**
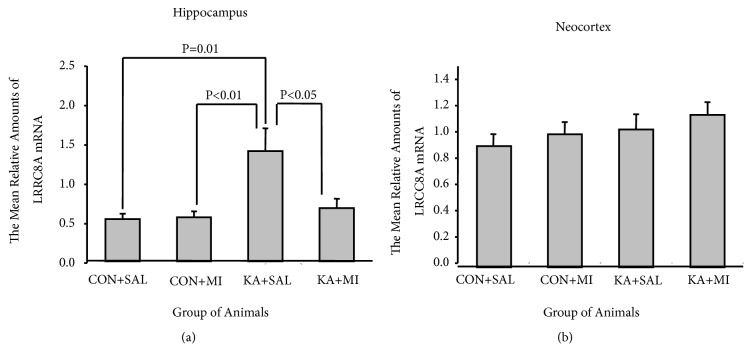
The mean relative amounts of LRCC8A mRNA in hippocampus (a) and in neocortex (b).

One-way ANOVA revealed that experimental treatment had significant effect on LRRC8A levels in the hippocampus (F3,35= 6.92 P=0.001). The mean relative amounts of LRRC8A mRNA were significantly higher in KA+SAL group as compared to other three groups (KA+SAL vs KA+MI T= 2.43 16 DF P =0.027; KA+SAL vs CON+SAL T= 3.06 16 DF P= 0.007 and KA+SAL VS CON+MI T=2.96 16 DF P = 0.009; Figure 5(a)). There was no difference between the CON+SAL, CON+MI, and KA+MI groups.

No significant difference was detected in LRRC8A mRNA levels between the groups in the neocortex (Figure 5(b)).

#### 3.2.3. mi-RNA Profiling

Changes in mi-RNA spectrum were studied in the hippocampus of three groups of rats: CON+SAL, KA+SAL, and KA+MI. Nearly 70 miRNAs displayed significant differences (p<0.05) between the groups to either direction (for full outcome of data analysis see Supplementary material: Supplementary Table S1-ANOVA Results and Table S2–S4 T-test results). Data on significantly differentially expressed miRNAs with normal signal are provided in Table 1, while data on significantly differentially expressed miRNAs with normal and low signal are provided in Supplementary Materials (Tables S1–S4).

KA+SAL and KA+MI groups differ significantly from each other by the levels of 22 miRNAs. Changes of 4 of them (marked by *∗∗*) are of the same pattern in KA+MI and in CON+SAL groups compared to KA+SAL.

To validate microarray experiments, we focused on those miRNAs that exhibited a minimal signal intensity (500) at least in one of the experimental group samples (see the brochure “Validation of miRNA Microarray Results with Real-Time QPCR” http://www.lcsciences.com/discovery/technical-bulletin-validation-of-mirna-microarray-results-with-real-time-qpcr/) and chose one mi-RNA with a normal signal of expression (rno-miR-6216), which displayed the most pronounced difference between KA+SAL and KA+MI groups. For quantitative miRNA analysis there are no well-accepted universal housekeeper miRNAs, since their expression patterns vary across the tissues. Normalization using endogenous control genes is currently the most accurate method to correct for potential differences in RNA input or RT efficiency biases (see the same brochure). Therefore from our miRNA profiling data we have chosen 2 miRNAs as housekeepers (rno-miR-23b-3p and rno-miR-361-5p) that had the lowest variance of expression with probability of p > 0.55.

Validation studies were performed on RNA fraction from hippocampus and neocortex from 4 groups of rats: CON+SAL, CON+MI, KA+SAL, and KA+MI.

### 3.3. Hippocampus

One-way ANOVA revealed that the effect of experimental treatment was significant in the hippocampus for both housekeeper miRNAs (for miR-361-5p F3,35=4.85, P=0.007, and for miR-23b-3p F3,35=4.04, P=0.015).

**Figure 6:**
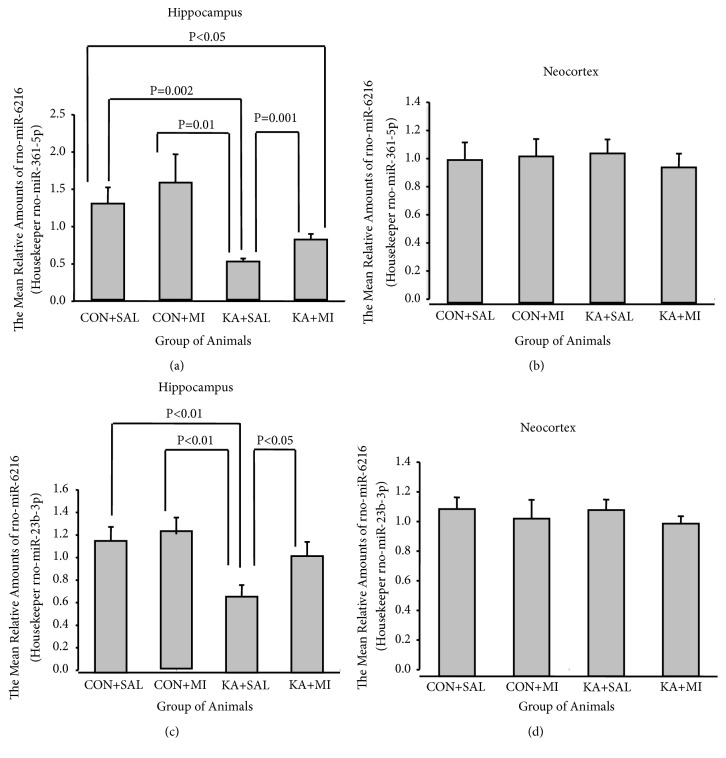
The mean relative amounts of rno-miR-6216 in hippocampus and neocortex. (a and b) With housekeeper: rno-miR-361-5p; (c and d) with housekeeper: rno-miR-23b-3p.

The mean level of miR-6216 in the hippocampus of KA+MI group was significantly higher as compared to KA+SAL group. This difference was valid when data were normalized for both housekeepers (see Figures 6(a) and 6(c), for miR-361-5p T = 3.93 16 DF P= 0.001 and for miR-23b-3p T =2.36, 16 DF P= 0.032). These results are in agreement with miRNA profiling data. The mean amounts of miR-6216 in CON+SAL and CON+MI groups are significantly higher as compared to KA+SAL group (see Figures 6(a) and 6(c), CON+SAL vs KA+MI for miR-361-5p T= 3.70 16 DF P= 0.002 and for miR-23b-3p T= 2.84 16 DF P= 0.012; CON+MI vs KA+MI for miR-361-5p T = 3.16 P = 0.006 DF = 16 and for miR-23b-3p T = 3.09 P= 0.007 DF = 16). The mean level of miR-6216 in CON+SAL was significantly higher as compared to KA+MI group (for miR-361-5p T= 2.21 P= 0.042 DF = 16). The difference between CON+MI and KA+MI group is significant only for one tailed test (for miR-361-5p T=2.03 P = 0.03 DF = 16). In case of miR-23b-3p as a housekeeper the difference between the CON+SAL, CON+MI, and KA+MI groups was not significant.

### 3.4. Neocortex

No significant changes were revealed by ANOVA (Figures 6(b) and 6(d)).

#### 3.4.1. HMGB1

**Figure 7:**
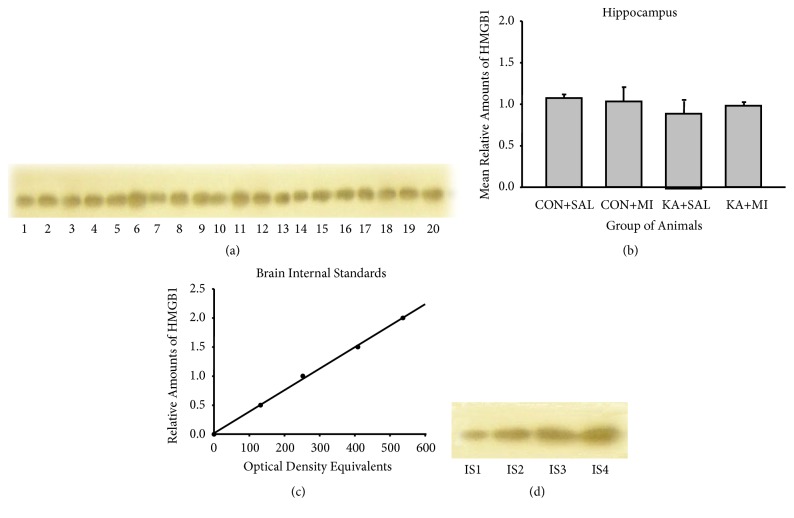
Sample film (a) and mean levels (mean ±standard error of the mean) (b) of hippocampus HMGB1 in different groups of rats. Each lane corresponds to one sample. Lanes 1-5 are from CON+SAL group; lanes 6-10 from CON+MI group; lanes 11-15 from KA+SAL group; and lanes 16-20 from KA+MI group. Calibration plots (lines fitted by linear least-squares regression) (c) and internal standards sample film (containing, respectively, 15, 30, 45, and 60 *μ*g protein) (d) for hippocampus HMGB1.

**Figure 8:**
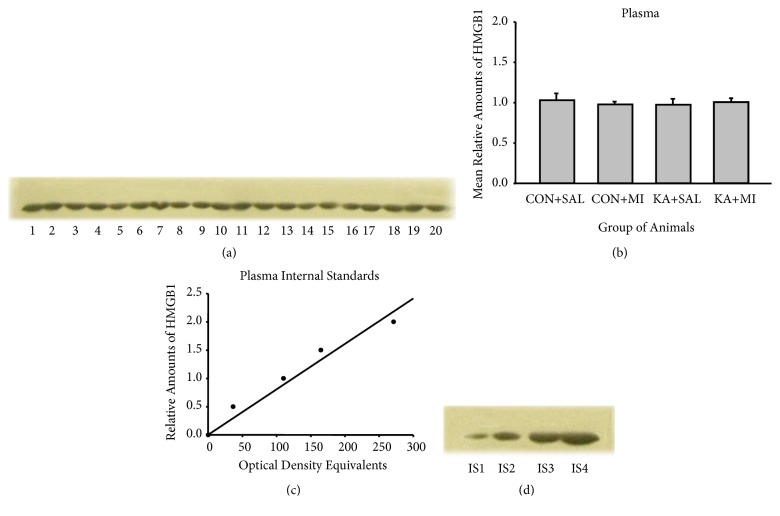
Sample film (a) and mean levels (mean ±standard error of the mean) (b) of plasma HMGB1 in different groups of rats. Each lane corresponds to one sample. Lanes 1-5 are from CON+SAL group; lanes 6-10 from CON+MI group; lanes 11-15 from KA+SAL group and lanes 16-20 from KA+MI group. Calibration plots (lines fitted by linear least-squares regression) (c) and internal standards sample film (containing, respectively, 15, 30, 45, and 60 *μ*g protein) (d) for plasma HMGB1.

Anti-HMGB-1 antibodies bind to a band of protein with molecular weight of 25 Kda (Figures 7 and 8). This band therefore corresponded to the expected size of the target protein.

Four standards (15, 30, 45, and 60 *μ*g of total protein) were applied to each gel. For these standards the optical densities of the HMGB1 immunostained bands were plotted against the protein amounts; least-squares regression showed a significant fit to a straight line for all these standards (Figures 7(c) and 8(c)).

No significant changes were found with one-way ANOVA in hippocampal, or plasma HMGB1 levels (Figures 7 and 8). No changes were observed in the neocortex too (data not shown here).

## 4. Discussion

The present results for the first time demonstrate that MI treatment have a long-term effect on KA-induced epileptogenesis: 4-week regular administration of MI, post-KA-induced SE, inhibits the spontaneous behavioural seizures with concomitant changes in molecular profile associated with epileptogenesis. This effect is maintained after termination of MI treatment.

### 4.1. Behaviour

The average number of SRS in the KA+MI group was much lower as compared to the KA+SAL group. This outcome was observed during the whole observation period for 8-weeks, as well as during the two separate periods: throughout actual MI treatment and after its termination. In addition, the total duration of SRS per animal was significantly shorter. Similar to the frequency, decrease in SRS duration was maintained after ceasing MI injections. Thus MI has a long-lasting effect on the process of epileptogenesis.

### 4.2. Molecular Changes

We have shown that 4-week MI treatment has significant effects on KA-induced molecular changes, which included SMIT and SWELL channel subunit expression, as well as miRNA spectrum.

#### 4.2.1. SMIT

We have found that the level of SMIT mRNA is significantly increased as compared to control groups in the hippocampus of KA+SAL and KA+MI groups. The level of SMIT mRNA in KA+SAL hippocampus is increased nearly 2 times as compared to the controls, whereas the increase in KA+MI is much stronger, exceeding control groups 6- and KA+SAL group 3 times. The upregulation of the SMIT mRNA in the hippocampus was also shown shortly after KA-induced SE in rats [13]. Are these changes directed to counteract epileptogenesis and severity of seizures? One of the major challenges in epilepsy research is to determine which molecular changes contribute to epileptogenesis after SE, which are compensatory and which are neutral [29]. Based on our behavioural analysis, together with the data from literature (see above), we argue that strong increase in the SMIT mRNA is most likely beneficial for promoting decrease in SRS frequency.

#### 4.2.2. LRRC8A

The only significant change in the amount of LRRC8A mRNA was observed in the hippocampus of the KA+SAL group. LRRC8A is one of the subunits of volume regulated anion channel (VRAC), and this channel is important for regulating cell size by transporting chloride ions and various organic osmolytes across the plasma membrane [16]. VRAC in the CNS is expressed both by neurons and by astrocytes, and in both cell types this channel is characterized with similar activation in hypoosmolar or excitotoxic conditions [17]. During intense neuronal excitation, such epileptiform bursts—massive influx of Na^+^, Ca^2+^, and Cl^−^—take place which leads to inward water flow, cellular swelling, and extracellular space reduction [30–32]. In line with this, reduced extracellular space (ECS) enhances neuronal excitability [33, 34]. Based on this, we could argue that VRAC activation might contribute to normalizing the ECS volume [35], thus counteracting epileptiform activity. To our knowledge, this is the first demonstration of increase of the VRAC channel component in post-SE epilepsy model.

Cellular swelling disrupts normal function of various enzymes [35]. Upregulation of osmoprotective gene expression and small organic osmolytes, including MI, can compensate for the cellular swelling. Taurine-transporter, tonicity-responsive enhancer binding protein, aldol-reductase, and SMIT belong to such osmoprotective genes and are upregulated 24h after SE [13, 36]. These upregulated osmolytes do not perturb the function of the intracellular enzymes. One could hypothesize that since animals in the KA+SAL group experience more frequent and longer seizures as compared to the animals in the KA+MI group, hippocampal cells are swelling more often, which in turn activates RVD response by VRAC, leading to release of solutes and balancing the osmotic gradient. This in turn would lead to normalizing the cell volume. The increased amount of LRRC8A would also facilitate this process.

#### 4.2.3. miRNA Spectrum

We demonstrate that 4 weeks of MI treatment have long-term influence on mi-RNA spectrum in the rat hippocampus. The miRNA hippocampal profile in KA+SAL and KA+MI groups significantly differ from each other by 22 miRNAs with normal signal expression and 28 miRNAs with low signal expression (see Table 1 and Supplementary Material–Tables S1–S4). The differences are in both directions: upregulation and downregulation of number of miRNAs. The separate comparisons of CON+SAL group with KA treated two groups also revealed significant differences: 9 mi-RNAs with normal expression are significantly different between the hippocampi of CON+SAL and KA+SAL groups and 21 mi-RNAs between the CON+SAL and KA+MI groups (Table 1, Tables S1–S4). For 4 miRNAs (rno-miR-181a-5, rno-miR-129-1-3p, rno-miR-664-3p, and rno-miR-150-5p, marked by *∗∗*) no difference is observed between CON+SAL and KA+MI groups, while significant differences are observed between CON+SAL and KA+SAL and KA+MI and KA+SAL groups. We have also studied the changes in the amount of miR-6216 using 2 endogenous housekeeper miRNAs. The data are consistent with miRNA profiling and convincingly indicate the significant differences between KA+SAL and KA+MI groups: namely, the amount of miR-6216 is higher in the KA+MI group. The amount of miR-6216 miRNA has been reported to increase in the rat cortex within 72 h after middle cerebral artery occlusion as compared to controls [37]. The targets of miRNA6216 include many important proteins such as protein tyrosine phospahatase receptor type R, mitogen activated protein kinase 8, and trafficking protein particle complex 6B (see http://www.mirdb.org/). Further studies are required to identify the expression patterns of these proteins.

Several miRNAs have been implicated in KA-induced seizures and KA-induced epileptogenesis [38–42]. To this end, seizure preconditioning was shown to result in upregulation of 25 mature miRNAs in the CA3 subfield of the mouse hippocampus, with the highest levels detected for miR-184 [41]. Silencing of microRNA-134 produces neuroprotective and prolonged seizure-suppressive effects [39], while miRNA-34a has been found to be upregulated during seizure-induced neuronal death [42]. However, the direct comparison of our data with the above results is not appropriate since different time points and models of KA delivery were used.

Taken together, all these data indicate that MI treatment alters the profile of KA-induced epileptogenesis as assessed by changes in levels of mi-RNAs that are important in regulation of posttranscriptional gene expression.

#### 4.2.4. HMGB1

No significant changes were found in HMGB1 protein in either hippocampus, or neocortex, or serum in any of the animal groups. We have measured the total amount of HMGB1 protein, which exists in various isoforms reflecting different pathophysiological processes. The disulfide isoform of HMGB1 is generated in the brain during oxidative stress and is implicated in seizures, cell loss, and cognitive dysfunctions [43]. Thus, we cannot exclude the possibility that despite the lack of changes in the total amount of HMGB1; some of the isoforms may still be changed under our experimental conditions.

MI is not only an important precursor to the biosynthesis of inositol phospholipids and inositol phosphates (compounds that play an important role in signal transduction) but also is a physiologically important osmolyte [reviewed in [44]]. The concentration of MI in the brain is in the range of 6 mM, which is 200-fold higher than cerebrospinal fluid concentration and 1400 fold higher than plasma concentrations in mammals [44]. Alterations in brain MI concentrations have been reported in a number of pathological conditions, including bipolar disorder, Alzheimer disease, and Down syndrome [44, 45]. Intracerebral vascular or IP injections of MI increase latencies to lithium-pilocarpine induced seizures in rats [46, 47]. Lithium is an uncompetitive inhibitor of inositol monophosphatase (IMPase) (enzyme producing MI) causing concomitant decrease of free MI. The pretreatment of animals with lithium and depletion of MI enhances pilocarpine induced SE [Inositol depletion hypothesis [48]]. In line with these observations, Na+/MI cotransporter was shown to be upregulated in the hippocampus shortly after IP administered KA-induced SE [13]. Moreover, increased levels of MI have been reported in seizure focus of TLE patients, whereas areas of seizure spread exhibited decreased levels of MI [49]. Mutations in IMPase-2 gene are associated with susceptibility to febrile seizures [50].

## 5. Conclusion

In conclusion, our data could open the novel perspectives in MI treatment as a possible preventive strategy in epilepsy by altering the progress of epileptogenesis that warrants further translational studies on MI-based approaches

## Figures and Tables

**(a):** 

mi-RNA	Log2 (KA+SAL/CON+SAL)	P value
rno-miR-494-3p*∗*	1.03	0.0053
rno-miR-100-5p	0.29	0.0083
rno-miR-582-5p*∗*	0.41	0.012
rno-miR-181a-5p*∗∗*	-0.41	0.020
rno-miR-652-3p*∗*	-0.56	0.024
rno-miR-28-5p	0.70	0.032
rno-miR-129-1-3p*∗∗*	0.99	0.037
rno-miR-664-3p*∗∗*	0.83	0.039
rno-miR-150-5p*∗∗*	0.27	0.0485

**(b):** 

mi-RNA	Log2 (KA+MI/CON+SAL)	P value
rno-miR-27a-3p*∗∗∗*	0.8	0.0012
rno-miR-135a-5p	2.28	0.0014
rno-miR-582-5p*∗*	0.61	0.0023
rno-miR-341*∗∗∗*	1.49	0.0033
rno-miR-329-3p*∗∗∗*	-0.7	0.0072
rno-miR-494-3p*∗*	1.16	0.0080
rno-miR-181d-5p	0.96	0.0134
rno-miR-543-3p	-0.43	0.0168
rno-miR-137-3p	0.70	0.0185
rno-miR-27b-3p*∗∗∗*	0.26	0.0197
rno-miR-434-3p	-0.53	0.0218
rno-miR-384-3p	0.51	0.0250
rno-miR-30a-5p*∗∗∗*	0.37	0.0287
rno-miR-195-5p	0.60	0.0297
rno-miR-376b-5p	-0.4	0.0338
rno-miR-433-3p	-0.57	0.0373
rno-miR-187-3p	-0.82	0.0379
rno-miR-138-5p	-0.68	0.0383
rno-miR-652-3p*∗*	-0.48	0.0410
rno-let-7a-1-3p*∗∗∗*	0.85	0.0412
rno-miR-485-5p *∗∗∗*	-0.84	0.0438

**(c):** 

mi-RNA	Log2 (KA+MI/KA+SAL)	P value
rno-miR-329-3p*∗∗∗*	-0.89	0.0015
rno-miR-341*∗∗∗*	1.52	0.0028
rno-miR-352	-0.64	0.0055
rno-miR-434-5p	-0.42	0.0068
rno-let-7e-5p	-0.71	0.0071
rno-miR-181a-5p*∗∗*	0.29	0.0078
rno-miR-126a-3p	0.33	0.0106
rno-let-7d-5p	-0.57	0.0157
rno-miR-30a-5p*∗∗∗*	0.32	0.0165
rno-miR-485-5p*∗∗∗*	-0.73	0.0178
rno-miR-494-5p	1.32	0.020
rno-miR-27b-3p*∗∗∗*	0.33	0.0267
rno-miR-3596a	3.27	0.0290
rno-miR-27a-3p *∗∗∗*	0.51	0.0381
rno-miR-6216	2.95	0.0357
rno-miR-664-3p*∗∗*	-0.69	0.0373
rno-miR-181c-5p	1.44	0.0388
rno-miR-185-5p	-0.29	0.0442
rno-let-7a-1-3p*∗∗∗*	0.78	0.0452
rno-miR-129-2-3p	-0.90	0.0453
rno-miR-129-1-3p*∗∗*	-1.19	0.0468
rno-miR-150-5p*∗∗*	-0.33	0.0491

## Data Availability

All miRNA profiling data are available as Supplementary Material, Tables S1–S4. Other experimental data will be available upon request.

## Supplementary Materials

Supplementary Materials Table-S1. miRNA profiling data–ANOVA results for the hippocampus miRNA of three groups of rats: CON+SAL, KA+SAL, and KA+MI, Table-S2. Comparison of miRNA expression between two groups of rats: CON+SAL and KA+SAL (t-test), Table-S3. Comparison of miRNA expression between two groups of rats: CON+SAL and KA+MI (t-test), Table-S4. Comparison of miRNA expression between two groups of rats: KA+SAL and KA+MI (t-test).
